# Accuracy of Cytology Specimen and Needle Core Biopsies for Detection of *KRAS* Mutation in Non-Small Cell Carcinoma: Comparison With Resection Specimen

**DOI:** 10.4021/wjon416w

**Published:** 2011-12-19

**Authors:** Ismatun Swati, Shengle Zhang, Jamie Tull, Kamal K Khurana

**Affiliations:** a Department of Pathology, State University of New York Syracuse, New York, 13210 USA

**Keywords:** KRAS mutation, Lung cancer, Non-small cell carcinoma, Cytology

## Abstract

**Background:**

Recent studies have shown that *KRAS* mutations are negative predictors of benefit from both adjuvant chemotherapy and anti-EGFR directed therapies for non-small cell lung carcinoma (NSCLC). Needle core biopsy, cytology specimen and resected tissue have all been used for *KRAS* mutational analysis of malignant lung tumors. However, studies validating the correlation between needle core biopsy/cytology specimen and resected tissue, histologic reference standard for KRAS mutational analysis are lacking. We retrospectively compared the *KRAS* mutation detection on cytology specimen or needle core biopsy with corresponding resected malignant neoplasm of lung, the histologic reference standard for mutational analysis.

**Method:**

Twenty-seven samples including 8 cell blocks, 9 cytology smears and 10 needle core biopsies, and corresponding 22 resected malignant tumor of lung were correlated for *KRAS* mutational analysis. In cases where cell block material did not correspond with results on resected specimen, cytology smears of corresponding cases were microdissected for isolation of DNA.

**Results:**

The needle core biopsy specimens and the corresponding surgical resections showed 100% concordant results for *KRAS* mutational analysis. *KRAS* mutation was detected in 4 out of 8 cell blocks, compared to 7 out of 8 corresponding surgical resections. Low cellularity (2 cases) and failure to retrieve DNA (1case) resulted in lack of correlation in 3 cases with cell blocks. However, cytology smears in these 3 cases confirmed the *KRAS* mutation noted in corresponding surgical resections. Overall concordance between cytology smears and corresponding surgical resections was 89% (8 of 9 cases). *KRAS* mutation was detected in 1 of the 9 cytology smears and was lacking in corresponding surgically resection.

**Conclusion:**

Cytology specimen and needle core biopsies provide adequate material for *KRAS* mutational analysis. Excellent mutational analysis concordance between cytology specimen/needle core biopsies and resected tumor suggests that predictive marker based therapeutic decision need not shift to more invasive surgical procedures.

## Introduction

Non-small cell carcinoma that harbor an activating mutation in the epidermal growth factor receptor (EGFR) kinase domain are associated with sensitivity to tyrosine kinase inhibitor (TKIs) [1, 2]. On the other hand, mutation in Kirsten RAS (*KRAS*), which encodes a GTPase, downstream of EGFR pathway, is associated with primary resistance to TKIs [3]. The *KRAS* gene encodes the human cellular homolog of a transforming gene of the Kirsten rat sarcoma-2 virus [4]. EGFR mutations are more commonly found in tumors from patients who never smoked cigarettes [2], while *KRAS* mutations are present in those with significant tobacco exposure [3]. *KRAS* mutational analysis is critical for predicting anti-EGFR therapeutic response in lung adenocarcinoma. Sufficient and reliable tissue samples are essential for mutational analysis.

Needle core lung biopsies and cytology specimen are most often the first sample to be obtained for diagnosis of lung primary and work up advanced cases of lung cancers, respectively. Hence an ideal approach would be to perform molecular tests on cytology specimen or needle core biopsy to optimize treatment. Although *KRAS* mutational analysis has been performed using cytology specimen and needle core biopsies, most of these studies lack comparisons to resected specimen for validation [5-9]. Concordance between cytology and resected specimen for mutational analysis is unknown. Recently, Solomon et al compared core biopsies and corresponding surgical resection and showed that core biopsies can yield sufficient and reliable samples for *KRAS* mutation analysis [9].

The purpose of this study was to retrospectively compare the accuracy of cytology specimen or needle core biopsy obtained prior to resection with that or resected lung tumor specimen, the histologic gold standard, for *KRAS* mutational analysis.

## Material and Method

This was a retrospective study and received exemption for IRB approval.

### Surgical specimen

Computer search at our institution from January 2001 to December 2008 yielded 22 lung adenocarcinoma resections that also had available cytology specimen or core needle biopsy of lung tumor prior to resection. Core biopsies of lung tumor were available in only 10 cases. Formalin-fixed paraffin embedded (FFPE) tissue block of resected tumor, and core biopsies were used for isolation and amplification of DNA. The percentages of tumor cells in the core biopsies and surgical resection specimen selected for molecular study ranged from 10% to 70%. Direct sequencing was applied to detect *KRAS* mutations in codon 12 and 13.

### Cytology specimens

Seventeen cytology specimen including 8 FFPE cell blocks and 9 Diff Quik (DQ)-stained smears were available on 12 patients with lung adenocarcinoma who had also undergone surgical resection. Procedures that were performed on these 12 patients to procure cytology specimen included CT-guided FNA of lung tumor [8], Transbronchial FNA [3] and bronchial washing [1]. All the hematoxylin and eosin (H&E)-stained slides from the FFPE cell blocks were reviewed by the pathologist to quantify the tumor cells. The percentages of tumor cells in the specimen selected for molecular study ranged from 2 to 40%. In the samples where tumor cells were scanty, the percentage was determined by counting the cells in representative high power field. The tumor foci were marked on the H&E- stained slides obtained from the FFPE cell block and used as a guide to scrape the tumor cells from unstained slide. In the cases of direct smears, the DQ slides were marked and tumor cells were isolated using pin point isolation technique that resulted in precise isolation of tumor cells (greater than 90%).

### Genomic DNA extraction

Total DNA was extracted from FFPE sections and cytology stained smear slides using Zymo’s Pinpoint Slide DNA Isolation System (Orange, CA). Sections of FFPE tissue were mounted on slides and upon a pathologic exam; areas of only tumor were outlined and collected using a scalpel. Cytology stained smear slides were placed in xylene for 3 - 6 days to float off coverslip and only stained tumor cells were collected using a needle using a needle under the microscope. DNAs from all samples were extracted per manufacturer’s instructions.

### COLD PCR

DNAs extracted were tested by co-amplification-at-lower denaturation-temperature PCR (COLD PCR) based on a previously described method by Z Zuo et al [10]. Briefly, COLD PCR was performed in a 50 µL of reaction mixture containing 1 µL of extracted DNA, 0.2 µmoL/L each primer (Table 1), 250 µmoL/L of dNTP mix, 2.5 mmoL/L MgCl2, 1x PCR buffer, and 1U AmpliTaq Gold (Applied Biosystems, Carlsbad, CA). The reaction mixture was run as follows: 95 °C for 10 minutes, 10 cycles of 95 °C for 15 seconds, 57 °C for 30 seconds, 72 °C for 1 minute; 72 °C for 7 minutes, 95 °C for 2 minutes; 40 cycles of 95 °C for 15 seconds, 70 °C for 8 minutes, 80 °C for 3 seconds, 55 °C for 30 seconds, 72 °C for 1 minute on a GeneAMP PCR system 9700 (PE Applied Biosystems, Foster City, CA). A positive control with a known *KRAS* mutation and a negative control of non-template sample were included for each PCR. These PCR products were then electrophoresed on polyacrylamide Ready Gels of 10% TBE (Bio-Rad, Hercules, CA), stained with ethidium bromide and 1 band of 98 bp was expected.

**Table 1:** Primer Used for COLD PCR

Name	Sequence
*KRAS-ZuoF^10^	5'-TATAAACTTGTGGTAGTTGG-3'
*KRAS-ZuoR^10^	5'-ATTGTTGGATCATATTCGT-3'

All Sequences Were Synthesized by Integrated DNA Technologies Inc. (Coralville, IA)

*F for forward and R for reverse.

### Purification and DNA Sequencing

Centri-sep spin columns (Princeton Separations, Adelphia, NJ) were used to purify PCR products per manufacturer's instructions. Purified products were labeled for sequencing using BigDye Terminator version 1.1 cycle sequencing kit (Applied Biosystems, Foster City, CA) according to manufacturer’s instructions. Products were then subjected to another centri-sep spin column purification for removal unincorporated dye labels and then run on an ABI 310 Genetic Analyzer running Sequencing Analysis Software version 5.2 (Applied Biosystems, Foster City, CA). Products were run in duplicates and with a positive control with a known *KRAS* mutation.

## Results

The test results of *KRAS* mutation on 10 core biopsies and corresponding surgical resection specimen are shown in Table 2. *KRAS* mutations were detected in 7 out of 10 core biopsies and their corresponding resection specimen. In 3 cases, no mutation was detected in both core biopsy and resection specimen. Surgical biopsy specimen showed 100% concordance with the corresponding resection specimen.

**Table 2:** Detection of *KRAS* Mutation in Needle Biopsies and Corresponding Surgical Resections

Case ID	Biopsy		Surgical		Concordance
	% tumor	Result (base)	% tumor	Result (base)	
1	30	G12V (GTT)	50	G12V (GTT)	Yes
2	25	G12D (GAT)	70	G12D (GAT)	Yes
3	40	G12C (TGT)	40	G12C (TGT)	Yes
4	30	G12C (TGT)	30	G12C (TGT)	Yes
5	30	No mutant	40	No mutant	Yes
6	< 10	G12S (AGT)	70	G12S (AGT)	Yes
7	50	No mutant	60	No mutant	Yes
8	40	G12A (GCT)	30	12A (GCT)	Yes
9	50	No mutant	60	No mutant	Yes
10	10	G12A (GCT)	25	12A (GCT)	Yes

The results of *KRAS* mutations tests on 8 FFPE cell block (cytology) specimen and the corresponding surgical resection cases were shown in Table 3. Status of *KRAS* mutation in 5 out of 8 (63%) cell blocks showed concordance with their corresponding surgical resection. However, *KRAS* mutation detected in 3 surgical resection specimen was not detectable in the corresponding cell blocks.

**Table 3:** Detection of *KRAS* Mutation in Cytology Cell Blocks and Corresponding Surgical Resections

Case ID	Cytology Cell Block		Surgical		Concordance
	% tumor	Result (base)	% tumor	Result (base)	
1	< 10	G12V (GTT)	50	G12V (GTT)	Yes
2	15	G12C (TGT)	40	G12C (TGT)	Yes
3	30	G12D (GAT)	60	G12D (GAT)	Yes
4	30	G12V (GTT)	60	G12V (GTT)	Yes
5	20	No mutation detectable	35	G12C (TGT)	No
6	< 2	No mutation detectable	40	G12C (TGT)	No
7	2	No mutation detectable	20	G12D (GAT)	No
8	60	No mutation	60	No mutation	Yes

**Figure 1:**
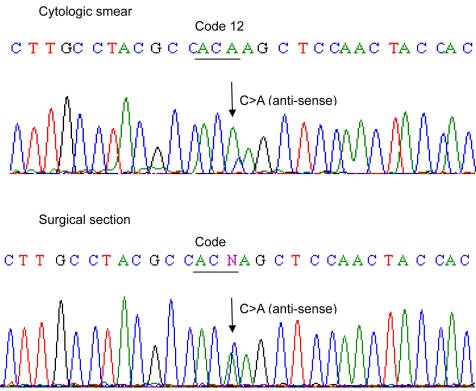
Example (case 6, Table 3) of DNA sequence (anti-sense) of KRAS mutation in cytologic smear and corresponding surgical section. KRAS mutation in code 12 (G12C) were identified in both cytologic and corresponding surgical specimen. Please note that mutant peak (C > A) is higher in cytologic smear than in surgical resection.

The results *KRAS* tests on 9 cytology smears and the corresponding resection specimen are shown in Table 4 These 9 cytology smears included 3 cytology cases (Cytology case ID# 5, 6, 7, Table 3) that were initially tested on cell block and did not correlate with the surgically resected tissue for *KRAS* mutation analysis. Eight of 9 cytology smears showed the same results as in the corresponding surgical resections including 3 mutants which were not detected in the corresponding cell blocks, with 89% concordance (Fig. 1). *KRAS* mutation was detected in one cytology smear and was not detected in the resected surgical specimen.

**Table 4:** Detection of *KRAS* Mutation in Cytology Smears and Corresponding Surgical Resections

Case ID	Cytology Smear		Surgical		Concordance
	% tumor^1^	Result (base)	% tumor	Result (base)	
1	N/A	No mutant	30	No mutant	Yes
2	N/A	G12D (GAT)	60	G12D (GAT)	Yes
3	N/A	G12D (GAT)	30	G12D (GAT)	Yes
4	N/A	G12V (GTT)	25	G12V (GTT)	Yes
5	N/A	G12C (TGT)	35	G12C (TGT)	Yes
6	N/A	G12C (TGT)	40	G12C (TGT)	Yes
7	N/A	G12D (GAT)^2^	20	G12D (GAT)	Yes
8	N/A	G12D ( GAT)	35	G12D ( GAT)	Yes
9	N/A	G12F (TTT)	40	No mutation	No

^1^Not applicable since the tumor cells are isolated on smears with pinpoint technique; ^2^Small mutation peak at code 13 (GAC) as well

## Discussion

With advent of novel treatment modality for NSCLC, molecular testing for EGFR and *KRAS* mutations is of increasing clinical importance in daily clinical practice. Evaluation of *KRAS* mutational status is critical because *KRAS* is not only an important downstream step of EGFR but also its mutations is related with resistance to EGFR-targeted therapy [11-13].

Image guided lung core biopsy or cytology specimen (CT-guided FNA, transbronchial FNA and bronchial brushing and washings) represent minimally invasive procedures for histologic diagnosis staging of lung adenocarcinoma. Comparison of adequacy of these procedures with adequacy of surgically resected specimen, the histologic reference standard, for mutational analysis of tumors of the lung has not been sufficiently investigated. Hence it is uncertain if reliable material can be procured from these procedures for mutational analysis.

In our study we demonstrated 100% agreement between image guided core needle biopsy and surgical resection specimen for *KRAS* mutational analysis. Similar results were shown Solomon et al with an 89% (16 of 18 cases) concordance for mutational analysis between the two types of specimen (image guided core needle biopsy versus surgical resection specimen) [9]. Lack of concordance in 2 (11%) of their cases was attributed to unsatisfactory image core needle biopsy specimen.

Studies describing the use of routinely collected cytology specimen, such as smears or cell blocks for *KRAS* mutational analysis of lung tumors are few. Rekhtman et al demonstrated that of the 128 cytologic specimen comprising of cell blocks 98% were suitable for molecular analysis and revealed *KRAS* mutations in 25 (20%) cases [6]. Recently Billah et al also showed that *KRAS* mutation in 23.6% (41 of 174) of cytologic specimen of lung tumor [7]. Feasibility of fine needle cytological aspirates for *KRAS* mutational analysis has also been shown by Schuurbiers et al [8]. However, none of these studies have compared the *KRAS* mutational result of cytology specimen with resected lung tumor. Lack of such comparison raises a possibility that in cases with lack of detection of *KRAS* mutation on cytology specimen may be secondary to tumor heterogeneity and may represent a false negative.

In order to ensure the reliability of cytology samples for mutational analysis we paired both cell blocks and cytology smears with corresponding resected lung tumor tissue for *KRAS* mutational analysis. In our study we demonstrated 63% agreement in results for mutational analysis between cell blocks and surgically resected specimen. Lack of correlation in 3 (37%) of our cases could be attributed to low cellularity in cell blocks (2% tumor cells) in 2 cases and failure to retrieve DNA in one case. However, when we used the cytology smears of corresponding cell block cases, we achieved a 100% agreement between cytology specimen and resected lung tumor for *KRAS* mutational analysis.

Comparison of cytology smears with resected tumor specimen showed that 89% (8 out of 9) were in agreement for *KRAS* mutational analysis. Interestingly, *KRAS* mutation (G12F) was detected in one cytology smear (case 9, Table 3) that was not noted in resected lung tumor. This may be attributed to tumor heterogeneity and/or different percentage of tumor cells. The ease with which tumor cells can be selectively scraped from the smears reduces normal cell contamination during DNA extraction and therefore enhances tumor cell population as opposed to microdissection of tumor in FFPE where the stromal cells and inflammatory cells may reduce the percentage of tumor cells. Percentage of tumor cells isolated in the discrepant case was greater than 90% in cytology smear versus 40% in the resected lung tumor. G12F with two mutations in code 12 (GGT > TTT) is a rare variant of *KRAS* mutations, and has been previously reported [14].

Our study is limited by its small sample size. Nevertheless, excellent agreement with resected lung tumor specimen suggests that core biopsies as well as cytologic specimen derived from CT-guided, endoscopic bronchial ultrasound guided, trans-bronchial FNAs and body fluids yield adequate and reliable material for *KRAS* mutational analysis. Our data also shows that in cases of low cellularity in tumor cell block material, cytology smears are better than cell block for *KRAS* mutational analysis.

The disadvantage of use of direct smear for molecular studies is that diagnostic material is not recoverable once it is used for molecular study. If cytology smears are to be used for DNA retrieval, it is necessary to ensure there are enough slides available so that the entire diagnostic material with morphologic details is not lost.

In summary, we have demonstrated that the standard molecular technique of detecting *KRAS* mutation in lung tumor can be adequately applied on cytology specimen and core needle biopsies. *KRAS* mutation analysis on cytology specimen and core needle biopsy is highly accurate and comparable with that of resected lung tumor. The need for more invasive procedures to obtain sufficient tumor tissue for molecular test could thus be obviated. Presence of *KRAS* mutation of cytology smears and lack of detection in corresponding resected tumor in one of our cases suggests that cytology smears may be more suitable and sensitive source for *KRAS* mutational analysis. However more validation studies comparing mutational analysis on cytology smears and resected lung tumor on larger scale are needed.

## References

[1] Gazdar AF (2009). Activating and resistance mutations of EGFR in non-small-cell lung cancer: role in clinical response to EGFR tyrosine kinase inhibitors. Oncogene.

[2] Pao W, Miller V, Zakowski M, Doherty J, Politi K, Sarkaria I, Singh B (2004). EGF receptor gene mutations are common in lung cancers from "never smokers" and are associated with sensitivity of tumors to gefitinib and erlotinib. Proc Natl Acad Sci U S A.

[3] Pao W, Wang TY, Riely GJ, Miller VA, Pan Q, Ladanyi M, Zakowski MF (2005). KRAS mutations and primary resistance of lung adenocarcinomas to gefitinib or erlotinib. PLoS Med.

[4] Barbacid M (1987). ras genes. Annu Rev Biochem.

[5] Boldrini L, Gisfredi S, Ursino S, Camacci T, Baldini E, Melfi F, Fontanini G (2007). Mutational analysis in cytological specimens of advanced lung adenocarcinoma: a sensitive method for molecular diagnosis. J Thorac Oncol.

[6] Rekhtman N, Brandt SM, Sigel CS, Friedlander MA, Riely GJ, Travis WD, Zakowski MF (2011). Suitability of thoracic cytology for new therapeutic paradigms in non-small cell lung carcinoma: high accuracy of tumor subtyping and feasibility of EGFR and KRAS molecular testing. J Thorac Oncol.

[7] Billah S, Stewart J, Staerkel G, Chen S, Gong Y, Guo M (2011). EGFR and KRAS mutations in lung carcinoma: molecular testing by using cytology specimens. Cancer Cytopathol.

[8] Schuurbiers OC, Looijen-Salamon MG, Ligtenberg MJ, van der Heijden HF (2010). A brief retrospective report on the feasibility of epidermal growth factor receptor and KRAS mutation analysis in transesophageal ultrasound- and endobronchial ultrasound-guided fine needle cytological aspirates. J Thorac Oncol.

[9] Solomon SB, Zakowski MF, Pao W, Thornton RH, Ladanyi M, Kris MG, Rusch VW (2010). Core needle lung biopsy specimens: adequacy for EGFR and KRAS mutational analysis. AJR Am J Roentgenol.

[10] Zuo Z, Chen SS, Chandra PK, Galbincea JM, Soape M, Doan S, Barkoh BA (2009). Application of COLD-PCR for improved detection of KRAS mutations in clinical samples. Mod Pathol.

[11] Shepherd FA, Tsao MS (2010). Epidermal growth factor receptor biomarkers in non-small-cell lung cancer: a riddle, wrapped in a mystery, inside an enigma. J Clin Oncol.

[12] Riely GJ, Marks J, Pao W (2009). KRAS mutations in non-small cell lung cancer. Proc Am Thorac Soc.

[13] Takeda M, Okamoto I, Fujita Y, Arao T, Ito H, Fukuoka M, Nishio K (2010). De novo resistance to epidermal growth factor receptor-tyrosine kinase inhibitors in EGFR mutation-positive patients with non-small cell lung cancer. J Thorac Oncol.

[14] Lopez-Crapez E, Mineur L, Emptas H, Lamy PJ (2010). KRAS status analysis and anti-EGFR therapies: is comprehensiveness a biologist's fancy or a clinical necessity?. Br J Cancer.

